# Food Literacy Scale: Validation through Exploratory and Confirmatory Factor Analysis in a Sample of Portuguese University Students

**DOI:** 10.3390/nu15010166

**Published:** 2022-12-29

**Authors:** Raquel P. F. Guiné, Sofia G. Florença, Graça Aparício, Ana Paula Cardoso, Manuela Ferreira

**Affiliations:** 1 CERNAS Research Centre, Polytechnic Institute of Viseu, 3504-510 Viseu, Portugal; 2 UICISA:E Research Centre, Polytechnic Institute of Viseu, 3504-510 Viseu, Portugal; 3 CIDEI Research Centre, Polytechnic Institute of Viseu, 3504-510 Viseu, Portugal

**Keywords:** food literacy, food knowledge, nutrition knowledge, healthy eating, food consumption, questionnaire survey, university students, structural equation model

## Abstract

Eating behaviors and healthy food choices are associated with food literacy, and they have a huge impact on one’s health status. For that reason, to increase food literacy is a way to effectively help individuals make appropriate choices that help maintain health and diminish the incidence of non-communicable diseases. The objective of this work was to test and validate a scale to assess food literacy. The validation was conducted on a sample of 924 Portuguese university students. The scale was composed of 50 items, which were submitted to exploratory and confirmatory factors analysis. The final validated scale corresponded to a second-order model with a global factor called “Food literacy”, which retained 26 items distributed by three factors: F1—literacy about the nutritional composition of foods (10 items), F2—literacy about labelling and food choices (7 items), and F3—literacy about healthy eating practices (9 items). The internal consistency of the scale is very high, with an alpha higher than 0.9, and the Pierson correlations between the three factors and the global are also higher than 0.9. In conclusion, the present scale has been validated and can therefore be utilized to measure food literacy.

## 1. Introduction

Nutrition is one of the factors that greatly affect human health. It has been shown that healthy growth and development are possible with a balanced diet, among other factors. A poor nutritional status can cause obesity and chronic diseases, and improper diets can be considered a risk factor for health, impacting many non-communicable diseases [1].

Literacy in healthy food was defined as the degree to which people have the ability to obtain, process, and understand basic nutritional information as well as the services they need in order to make decisions that are best suited to health [2]. Food literacy encompasses, therefore, not only the obtaining of information, but also the process by which this information becomes knowledge incorporated into the minds of the individuals that allows them to make adequate food decisions. Therefore, a consumer who is endowed with greater food literacy can engage in a more conscious and informed decision-making process, aimed at a better health status. On the contrary, a low food literacy level can originate wrong food choices, with a decisive impact on disease [2,3,4,5,6].

Individual perception of healthy eating is a complex construct and reflects personal, cultural, and social experiences, as well as the surrounding environment, the latter being a determinant of food choices [7]. Food is a manifestation of culture, of the history of each person and that of social groups, closely related with health and well-being, with the potential to prevent or trigger diseases [8,9,10].

Possessing food literacy allows individuals to have access to healthy foods, since nutrition and eating habits are some of the most basic factors that affect human health. Food literacy is implicit in the way that external factors influence a person’s development and in the application of knowledge, skills, and behaviors necessary for healthy eating. Food literacy among higher education students is not yet explored. However, higher education presents a unique opportunity to promote food literacy [11]. To measure food literacy is pivotal to investigate the degree of knowledge about food and its effects on human health. Therefore, the objective of this study was to validate a scale to measure food literacy, using for that purpose a sample of higher education students in Portugal.

## 2. Materials and Methods

### 2.1. Instrument and Data Collection

The questionnaire included a number of items, 50to be precise, aimed to evaluate food literacy, to be answered on a 5-point Likert scale. The items are related to different aspects associated with food composition and nutritional value, eating practices and food labelling, and different dimensions of literacy: to obtain information, to understand information and from it produce knowledge, and finally to apply that knowledge to practical life situations. The study and the questionnaire that served as basis was approved by the Ethics Committee of the Instituto Politécnico de Viseu (Reference nº 15/SUB/2020).

Data were collected through the application of questionnaires, to adult participants, who consented in the study after being informed about its purpose and terms, and who voluntarily completed the survey online from January to April 2021. All data were collected and treated guaranteeing anonymity.

**Figure 1:**
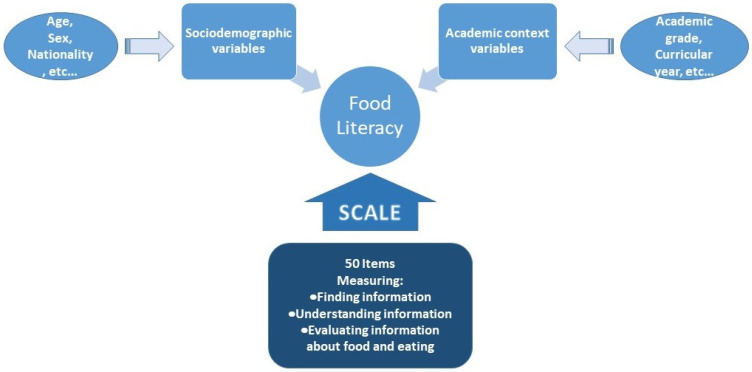
Schematic representation of the model to measure food literacy.

The questionnaire was developed in the ambit of the project “AppHealth: Empower to better live” approved by the Polytechnic Institute of Viseu and co-financed by *Caixa Geral de Depósitos*. The development of the questionnaire involved a series of meetings with different team members of the project. The formulation and choice of the items was based on the objectives of the project and followed some previous literature research on related questionnaires. The development of the data collection instrument (questionnaire) included the pre-test phase, aimed at verifying if the questions were perceptible for the participants and if they were adapted to actually measure the concepts that were idealized (Figure 1). The pre-test was achieved by administering the questionnaire through direct interview to a small number of 50 participants, following the recommended procedure by Hill and Hill [12]. This phase allowed for identifying which items might have been formulated in such a way that they were not so clear to the participants, and therefore, the resulting version of the questionnaire items was corrected according to the findings of the pre-test phase. The final version of the questionnaire was applied to a sample of 924 partic-ipants, with this number being higher than the minimum number of participants required for statistical validation recommended by Hill and Hill [12], which is 100 participants. Additionally, to guarantee practical validity of the scale, the number of participants should be five or six times higher than the number of items in the questionnaire. In this case, the number of items was 50; therefore, a minimum of 250–300 participants should have beeen used.

In the research we used a convenience sample due to the facility of recruitment and easy verification of will to participate. Although some disadvantages are pointed out to the use of convenience samples, it is also generally recognized that they are very useful for exploratory research [13,14,15]. Although, when using conventional samples, it is not required to conduct a formal calculation of the sample size, it is still a useful indicator to follow in those cases as well. Hence, an indicative sample size was obtained considering a 95% confidence interval, corresponding to a level of significance of 5% and a z score of 1.96 [16,17]. The Portuguese population in 2020 was 10.333 million people, of which approximately 4% were students attending higher education courses (university/polytechnic), or more precisely 396,909 [18,19]. Considering that we targeted only university students, the calculated minimum number of participants should be 384 [20,21,22]. According to the distribution by sex of the Portuguese Higher Education students in 2020, 182,178 were male and 214,731 were female [19]. Hence, using the same proportion, the minimum number of male participants in this survey should be 176 and female participants should be at least 208. Both these numbers were guaranteed and surpassed. Regarding the age, we targeted students attending university courses, so there was no upper or lower age limit, although the normal age of entering university is about 18 years in Portugal for a regular student finalizing secondary school and entering university right after that.

The data were collected using the internet platform Google Forms, following invitations sent through online tools such as e-mail and social networks. The inclusion criteria were as follows: (1) being a student at a Portuguese university/polytechnic institute, (2) being willing to take place in the survey voluntarily, and (3) having internet access and a computer or other device through which they could answer the questionnaire.

### 2.2. Data Analysis

It was carried out a psychometric study of the instrument used in order to verify the validity of the items. Item–total correlations were calculated to measure at which degree each of the items were correlated to the rest of the scale. The reliability of the scale and of its items was further studied through the analysis of internal consistency, as well as validity through the verification of the factor structure.

Cronbach’s alpha coefficient (α) measures the internal consistency of items; that is, through its values is possible to verify if the measurement instrument will always give the same data when applied to equal targets [23]. Its values vary between 0 and 1, and the internal consistency is greater the closer the statistic value is to 1. An instrument is classified as having appropriate reliability when α is at least 0.70 (and at most 0.95).

All data processing was performed using software for statistical treatment and structural equation modeling, namely SPSS (Statistical Package for the Social Sciences) and AMOS. For data analysis, descriptive statistics and analytical or inferential statistics were used. A level of significance of 5% was considered in all statistical analyses, and it was performed using the SPSS (version 28) software.

Measures of association, such as Pearson’s correlation coefficient, were also determined. This is represented by “r” and is a measure of the direction and degree with which two quantitative variables are linearly associated, assuming values between −1 and +1. The greater the value of r, absolute value, the greater the degree of linear association between the variables [24]. For interpretation of the strength of Pearson’s correlation coefficient, the folllowing were considered [25]:

- r < 0.2—very low correlation;
- 0.2 ≤ r ≤ 0.39—low correlation;
- 0.4 ≤ r ≤ 0.69—moderate correlation;
- 0.7 ≤ r ≤ 0.89—high correlation;
- 0.9 ≤ r ≤ 1—very high correlation.

To test the applicability of exploratory factor analysis, the data were evaluated for suitability through the Bartlett’s test of sphericity and the Kaiser–Meyer–Olkin (KMO) coefficient. The reference values of the KMO test, which vary between 0 and 1, relative to the adequacy of the factor analysis, are as follows: between 0.900 and 1.000—very good; between 0.800 and 0.900—good; between 0.700 and 0.800—average; between 0.600 and 0.700—reasonable; between 0.500 and 0.600—bad; and less than 0.500—unacceptable. Bartlett’s sphericity test is based on the Chi-square statistical distribution and, for the factor analysis method to be adequate, the significance (*p*) value of this test must be less than 0.05 [26].

Exploratory factor analysis was performed using the principal components analysis (PCA), based on the communalities. To extract the factors, the Varimax orthogonal rotation method was used. Eigenvalues greater than 1 and the slope scree plot were used to fix the number of factors extracted.

For confirmatory factor analysis, performed as previously mentioned with the software AMOS, there are several statistics to validate the model built, as indicated in Table 1, with the corresponding interpretation limits [27].

### 2.3. Sample Characterization

In this study, we used a non-probabilistic sample composed of 924 higher education students, from which 736 were women and 188 were men. The ages varied between a minimum of 18 years and a maximum of 57 years for women and a maximum of 70 years for men (Table 2). The average age of female participants was lower than male participants (21.89 ± 5.37 and 24.14 ± 8.17 years, respectively).

As shown in Table 3, most female participants were in the age group up to 19 years old (28.8%), while the majority of male participants were aged 22 years or over (9.3%). With respect to the course attended by the higher education students, most participants were from a Licence degree (62.1% women and 12.9% men), followed by Master degree (13.3% for women and 4.3% for men).

## 3. Results

### 3.1. Psychometric Study of the Scale

To assess validity of the scale and its items, internal consistency and correlations were used. Table 4 shows for each of the 50 items the statistics, as well as the values of the correlation of that item with the global scale and the Cronbach’s alpha coefficients. According to the scale, scores close to 4 mean more literacy, while scores close to 0 mean less literacy. Values between 4 and 5 correspond to absence of answer by the participants. Items whose main score corresponded to high literacy include numbers 1, 3, 8, 10 to 16, 22 to 26, 31 to 34, 39, 40, and 43 to 50. All other items correspond to average scores above 4, corresponding to the greater influence of items with score 5 = “I do not know”. Items with higher correlation with the global score of the scale are numbers 31, 37, 42, and 43, with values above 0.8. Furthermore, Table 4 reveals that the global alpha is between 0.980 and 0.981, depending on the item removed, corresponding to an appropriate internal consistency.

### 3.2. Exploratory Factor Analysis

The suitability of the data for application of factor analysis was confirmed, since the value of KMO was 0.966, thus corresponding to very good on the scale, and the value of significance of the Bartlett’s test of sphericity was significant (*p* < 0.0005, so below 0.05).

**Figure 2:**
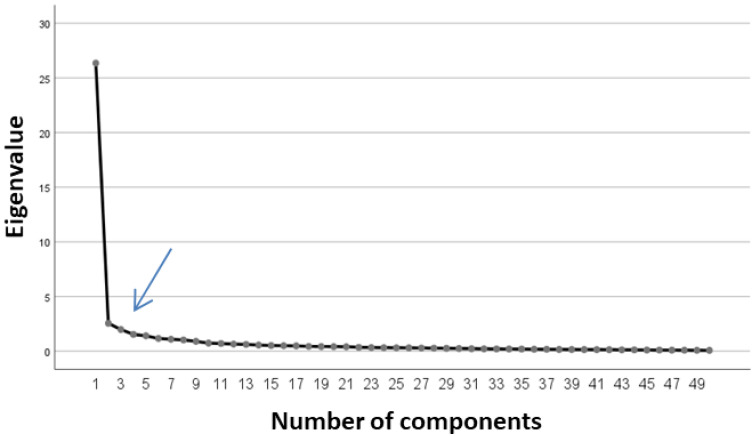
Scree plot for factor analysis through PCA with Varimax rotation.

We proceeded to exploratory factor analysis using the PCA method, with Varimax rotation. Eight factors were obtained that explained, in total, 74.19% of variance. However, based on the scree plot, the number of factors to retain should be three, according to the inflection point of the curve (Figure 2).

A forced analysis of three factors was, therefore, carried out, with a percentage of explained variance equal to 61.76%. Factor 1, called “literacy about the nutritional composition of foods”, consisted of 26 items and explains 27.14% of the total variance after rotation. It has an eigenvalue of 13.567. Factor 2, called “literacy about food labelling”, consisted of 11 items and explains 17.69% of the total variance after rotation. It has an eigenvalue of 8.845. Factor 3, called “literacy about healthy eating practices”, consisted of 13 items and has an explained variance percentage of 16.93%. It has an eigenvalue of 8.467.

Table 5 shows the items’ association with each of the factors, and the corresponding input weights. With respect to factor F1, the strongest items associated with it are numbers 27 (use of salt), 29 (control salt in diet), 30 (role of fats), 35 (role of carbohydrates), 36 (type of carbohydrates in diet), 37 (role of dietary fiber in health), 38 (benefits of fiber), 39 (benefits or drawbacks of excessive fiber), 41 (adequate protein consumption), and 42 (animal and plant protein sources), in all cases with values higher than 0.7. Similarly, the items that mostly contributed to the definition of factor F2 were numbers 48 (find information about the labels’ nutritional semaphore) and 49 (understand the nutritional semaphore). Finally, factor F3 was most strongly associated with items number 4 (understand information on healthy eating) and 9 (understand the information about diets).

### 3.3. Confirmatory Factor Analysis

After the exploratory factorial study of the scale, the hypothesized three-factor model was submitted to confirmatory factor analysis. All items showed symmetry and kurtosis values within the parameters considered normal, that is, less than 3 and less than 7, respectively, in absolute value, whose highest value was 1.989 for asymmetry and 5.315 for kurtosis.

For estimates and critical ratios, all items and corresponding factors are of highly significant statistical significance (represented as *** in Table 6), and we could keep all data. However, items with a factor loading of less than 0.40 were not maintained.

**Figure 3:**
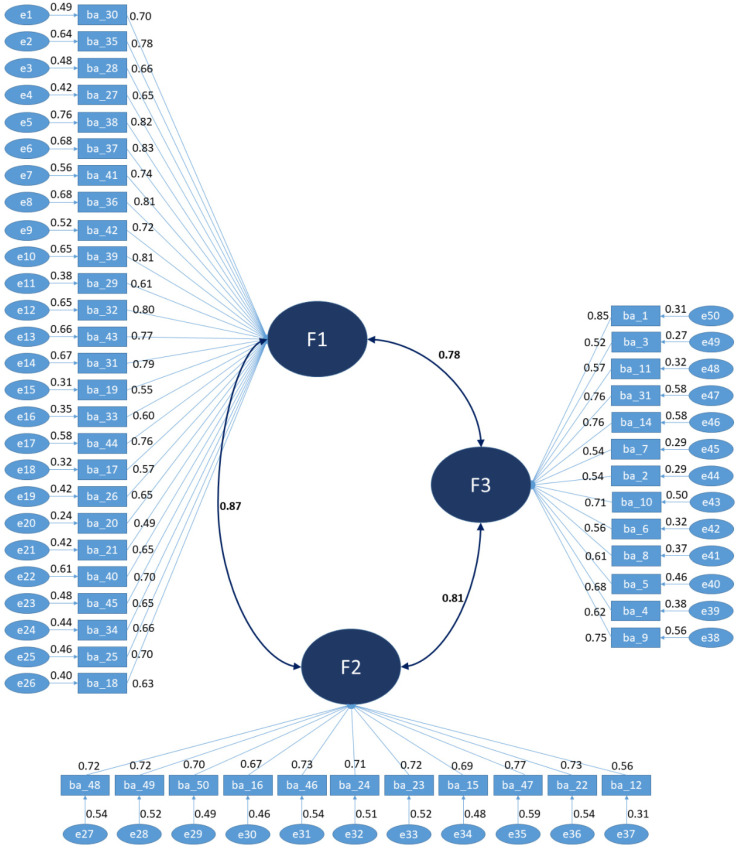
Initial model hypothesized with three factors.

Goodness and adjustment index values showed poor adjustment for Chi square/degrees of freedom (6.939), CFI (0.643), and GFI (0.554), and they were poor for RMR (0.071), unacceptable for RMSEA (0.113), and acceptable for SRMR (0.077). Figure 3 illustrates the hypothesized factorial model and the reliability and saturations of the items with the corresponding factors.

**Figure 4:**
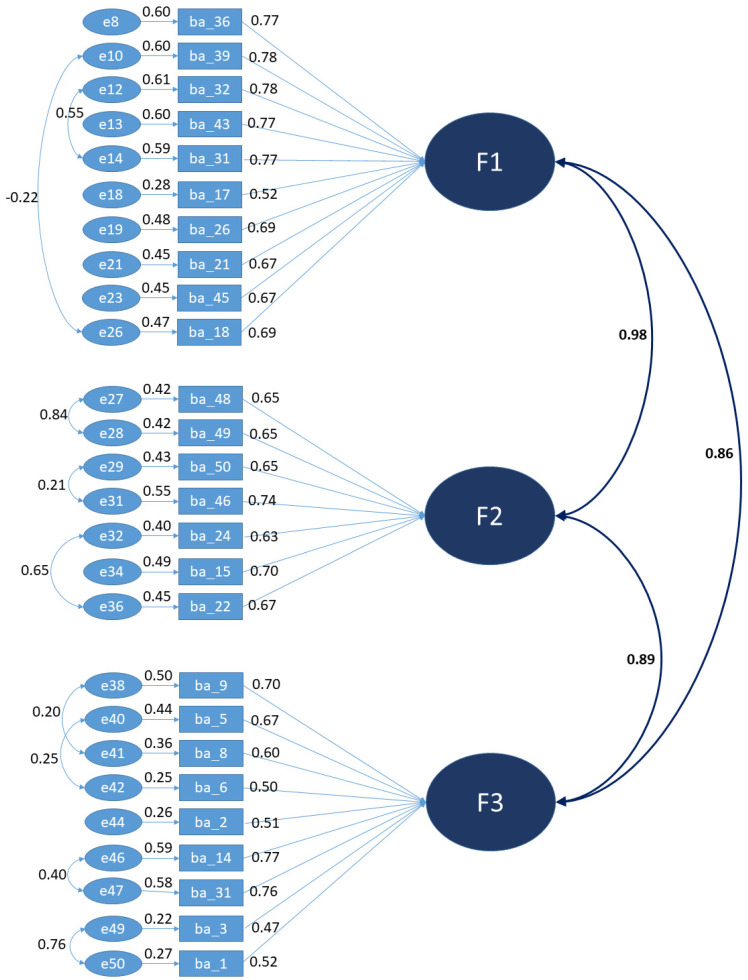
Re−specified model with modified indices by AMOS.

Then, the model was re-specified with the indices proposed by the software AMOS. By doing this, several items were eliminated due to multi-collinearity problems, with 24 items in total. Specifically, factor F1 was reduced from 26 to 10 items, factor F2 was reduced from 11 to 7 items, and factor F3 was reduced from 13 to 9 items. The utilization of factor analysis involves the analysis a variable number of items from the same questionnaire, and, although the more traditional approach of using linear factor model is suited to the analysis when using Likert-type items, it can produce dichotomous or ordered categorical variables [30]. Multi-collinearity comprises a relevant aspect both in multiple regression and generalized linear models and in structural equation modeling (SEM) or multilevel structural equation modeling (MSEM) [31]. The refined model, illustrated in Figure 4, presents the following indices of overall goodness of adjustment: Chi square/degrees of freedom = 3.702 (improvement for poor adjustment); CFI = 0.904 (improvement to good fit); GFI = 0.847 (maintained a poor adjustment); RMSEA = 0.076 (improvement for good fit); and RMR = 0.054 and SRMR = 0.055 (improvement for proper fit).

**Figure 5:**
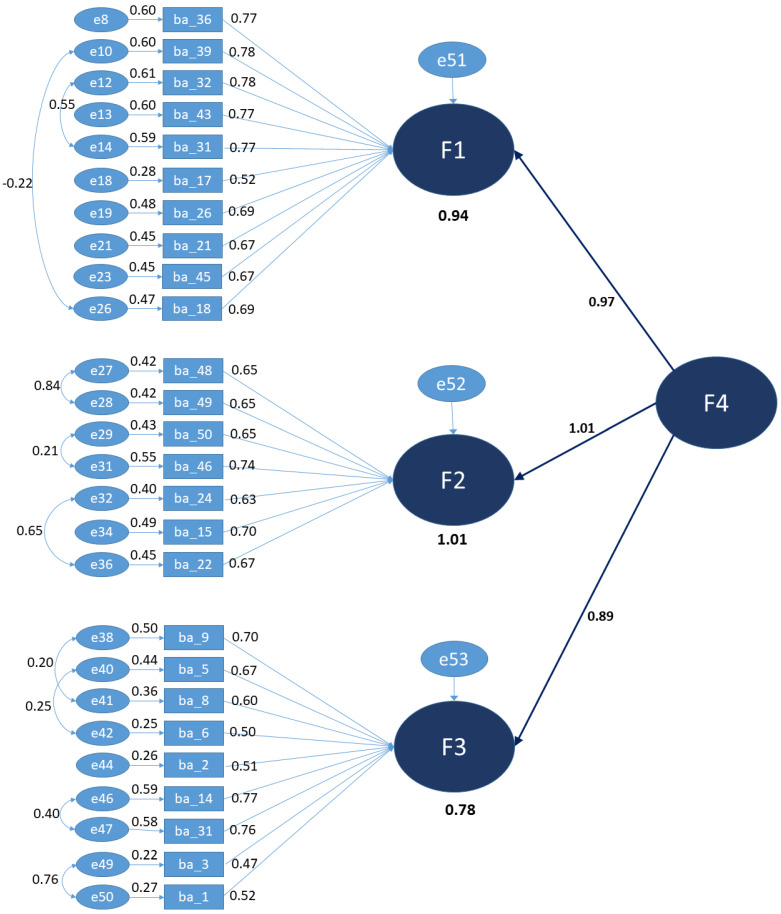
Final second-order model.

Correlational values are suggestive of a second-order model with a new global factor F4 that is herein called food literacy, which is presented in Figure 5.

In short, after reconstruction of the model through confirmatory factors analysis and structural equation modelling, three factors were formed that constituted the second-order model, whose global factor (Factor 4) was called food literacy. The designation and constitution of the factors are as follows:

- Factor 1: Literacy about the nutritional composition of foods. From factor F1, we excluded items 19, 20, 25, 27, 28, 29, 30, 33, 34, 35, 37, 38, 40, 41, 42, and 44, leaving only 10 items: 17, 18, 21, 26, 31, 32, 36, 39, 43, and 45.
- Factor 2: Literacy about labelling and food choices. Items 12, 16, 23, and 47 were excluded from factor F2, leaving only 7 items: 15, 22, 24, 46, 48, 49, and 50.
- Factor 3: Literacy about healthy eating practices. Items 4, 7, 10, and 11 were excluded from factor F3, leaving it to only be constituted by 9 items: 1, 2, 3, 5, 6, 8, 9, 13, and 14.

In Table 7, we present the description of each item of the three factors that were included in the final model. The scale, which contains 26 items in total, has an overall alpha of 0.962 or 0.961, depending on the item removed, and the scale’s internal consistency after confirmatory factor analysis is also shown in Table 7.

Table 8 presents the internal consistency by subscale (factors) after the confirmatory factor analysis of the scale. In factor F1, the item–total correlation oscillates between 0.653 in item 17 and 0.795 in item 31, with variabilities of 44.3% and 73.3%, respectively. All items have Cronbach’s alpha coefficients greater than 0.921 (good) with an overall alpha of 0.931. In factor F2, the item–total correlation has the lowest index in item 15 (r = 0.603) with a variability of 39.2% and the highest index in item 48 (r = 0.770) with a variability of 76.4%. All items have Cronbach’s alpha coefficients greater than 0.873 (good) with an overall alpha of 0.897. In factor F3, the item–total correlation has the lowest index in item 6 (r = 0.637) with a variability of 43% and the highest index in item 3 (r = 0.698) with a variability of 60.9%. All items have Cronbach’s alpha coefficients greater than 0.894 (good) with an overall alpha of 0.908.

The analysis is concluded by presenting the Pearson correlation matrix that was established with the scale factors and the global factor. Based on the analysis of Table 9, the subscales establish positive and significant correlations with each other, with the lowest being observed between factors F2 and R3 (r = 0.725) and the highest between factors F1 and F2 (r = 0.849). The correlations of the subscales with the global factor are higher, being above 0.900.

## 4. Discussion

### 4.1. Implications and Particularities of the Higher Education Students towards Food

The transition from secondary education to higher education is associated with many changes, in levels of social influence and surroundings, which can be a risk factor for unhealthy lifestyles. This transition leads to changes in food consumption, from skipping meals to eating nutritionally poor foods. Students, as a rule, have short schedules, which can contribute to them having a greater number of meals away from home, skipping meals, or adopting unhealthy eating practices, such as consumption of fast food and low consumption of fruits and vegetables. They end up developing poor eating habits, usually with a high intake of carbohydrates and saturated fat and low intake of vegetables and fruits, causing a nutritional deficiency of vitamins and fibers. Apart from the already discussed poor eating habits, the practice of skipping breakfast also negatively impacts the well-being of the students [32]. Florença et al. [32] carried out an investigation in which they studied eating habits in a sample of 670 students from higher education establishments in Central Portugal, finding that, interestingly, most students had satisfactory eating habits. A considerable percentage of the participants, 40.8%, had five meals a day, and only 6.6% of the participants indicated that they normally do not eat breakfast [32]. Periods of intense study and the engagement in a part-time job can result in a greater constraint in time management and increased levels of stress, leading to less careful food intake.

Students, when entering university, mostly go through moments of insecurity related to a new lifestyle. They also have positive expectations about their new life, which are almost always shared with family and friends, with a certain pride, putting pressure on young students, in addition to the pressure already created by living away from their usual family and friends [33]. Higher education students constitute “a risk group” for inadequate eating habits, weight gain, low levels of physical activity, and insufficient and poor-quality sleep hours [34]. Lifestyle influences the general state of health and may influence cognitive performance [35]. These are characterized by a sedentary lifestyle and excessive intake of foods with high energy density and alcohol and low intake of fruit and vegetables as well as water [11,35]. With regard to hydration, higher education students often replace water with sugary drinks, which, due to their high sugar content, can contribute to weight gain and development of chronic diseases, especially type 2 diabetes mellitus. Hydration is essential to ensure body homeostasis, so it seems to positively influence cognitive performance and attention [35]. Therefore, inappropriate food behaviors can lead to the development of chronic diseases in the long term and compromise their academic achievement.

Higher education students can be considered a risk group for inappropriate eating habits and weight gain, since the transition to higher education often represents a critical period with an impact on individuals’ eating habits. This period coincides with a period in which many young adults acquire greater freedom and independence, beginning to be responsible for choosing, buying, and cooking food. Thus, the promotion of adequate eating habits in these population groups is extremely important, and higher education institutions can play an active role in this area [34,36,37,38,39].

Higher education students mostly do not follow the Mediterranean Diet (MD), which is a food pattern characteristic of some Mediterranean countries, including Portugal. MD is abundant in minimally processed plant foods; rich in monounsaturated fat from olive oil; low in saturated fat, meat, and dairy products; and has proven an ideal nutritional model for cardiovascular health [40,41,42,43]. Based on a systematic review of the literature, evidence demonstrates that the traditional MD is associated with better cardiovascular health outcomes, including clinically significant reductions in the rates of coronary heart disease, ischemic stroke, and total cardiovascular disease [44,45,46]. The MD represents a prominent general dietary pattern in nutritional epidemiology that has been extensively studied, especially during the last two decades [47,48,49,50,51,52].

Higher education is considered a crucial period for students to develop healthy eating habits and adopt a healthy diet, which form a solid foundation for good health throughout life [1]. Kabir et al. [1] carried out a study in which they investigated the factors that influence eating behavior and food intake in Bangladeshi higher education students, using a qualitative approach, having carried out 25 interviews and 13 focus group discussions with students from several courses. The results reveal that students’ eating behavior and food intake are influenced by a variety of factors, namely: individual factors (cooking skills, food taste, food taboos, knowledge, and perceptions); social factors (peer influence and social norms); university-related factors (campus culture and exam frequency); and environmental factors (availability of cooking resources and facilities, as well as the price of food).

### 4.2. The Measurement of Food Lietracy

In the United Kingdom, a study called Sodexo University Lifestyle Survey [53] was carried out, with a target population of 2001 higher education students from all over the country, which aimed to understand what were the students’ needs as a way of providing them with better services and literacy in health. This study showed that less than half of the students (43.0%) ate breakfast every day, and 12.0% reported that they never had breakfast. It was found that 45.0% of the students did not have lunch at least once a week, with dinner being the most regular meal (80.0%). However, 81.0% stated that they made an effort to eat healthy; 51.0% of the students reported having drunk less than units of alcohol per week (equivalent to about 4.5 L of beer), with only 1.0% reporting that they drank more than 41 units per week (about 20 L) and 6.0% between 21 and 40 units.

Liao et al. [54] carried out a cross-sectional study that aimed to investigate the food literacy status in Taiwanese university students and assess the relationship between food literacy and healthy eating behaviors. Students were from six universities in Northern, Central, and Southern Taiwan, either national or private universities in each region. In total, 220 students from each university participated in the study. Data were collected using a self-assessment food literacy scale and questions about the frequency of practicing healthy eating behaviors and sociodemographic characteristics. The hierarchical regression results showed that food literacy explained 17.2% of the total variance of healthy eating behaviors of university students. It was shown that students had low food literacy, and there was a positive association between higher levels of literacy and healthier eating behaviors. In view of these results, the authors state that it is essential to promote healthy eating behaviors in higher education students [54].

The work by Kabir et al. [1] conducted with a sample of Bangladeshi higher education students suggests that students have an inadequate food intake, which can have a detrimental impact on their health, well-being, and academic performance. Therefore, the authors suggest interventions that empower students with more food literacy, providing nutritional information at various levels, which translates into benefits to promote healthy eating behavior and food intake among higher education students.

Zwierczyk et al. [55] conducted a validation of the Short Food Literacy Questionnaire based on a sample of 1286 Polish internet users. The authors used exploratory and confirmatory factor analyses (EFA and CFA, respectively), and applied the techniques by randomly dividing the data into two independent sets. Based on the values of internal consistency (Cronbach’s alpha over 0.8), the scale was validated with a three-factor structure as follows: (1) assessing information, (2) knowledge, and (3) information appraisal [55].

Luque et al. [56] performed a validation of the Self-Perceived Food Literacy Scale on a sample of 362 Spanish university students, mostly women, obtaining a five-factor model with good internal consistency (alpha close to 0.9). Besides CFA, external validity was also assessed, finding significant correlations between the variables [56].

Another important aspect of food literacy is knowing how to analyze food labels. These are intended to inform the consumer about the nutritional properties of a food, declaring its energy value and the main nutrients [57]. These labels must be presented clearly and with the correct specification of quantity, composition, and quality, as well as the risks they may present. This information is mandatory and contributes to the promotion of healthy eating [57]. It has been reported that Portuguese consumers generally read food labels and realize their importance, even though not fully understanding all the information contained in the label [58]. While being easier for them to understand the front-of-pack labelling, and most especially those presented through symbols/colors, they find it more difficult to consult the other bits of information provided in the labels [58]. In this way, it is necessary to better educate consumers, namely with respect to food literacy, and to give them the tools that allow for the proper interpretation of labels to make informed food choices [58].

It is known that the availability of clearly described food labels tends to increase credibility and security in relation to this information and allows the consumer to assess whether the product meets their nutritional and food needs [57]. However, most consumers, especially younger ones, do not show interest in this information, as they tend to be unaware of its importance [57]. Additionally, few can effectively understand and trust the information presented to them in food labels. Therefore, there is a need to develop simpler and clearer labels to consequently opt for healthier eating practices [57]. The nutritional semaphore (or traffic light labelling) meets these goals [59,60,61,62] and has been introduced successfully in several countries, including Portugal [63,64], although some argue that it is misleading, by being too simplistic and not providing all necessary information for a correct food choice, just like the other alternative, which is the nutri-score [65,66].

### 4.3. Limitations of the Study, Value, and Future Perspectives

The value of the present work is undoubtedly related to the statistical validation of the instrument, which will allow for evaluating the food literacy of groups of individuals with similar characteristics as those in the sample utilized. Specifically, this instrument is validated to apply to Portuguese higher education students. Nevertheless, the advanced statistical treatment performed can be an indicator that some extrapolation can also be valid under some circumstances. For example, it can be extended to participants in other countries, as long as they are also higher education students, given they particularities differentiating from other target groups, like general young adults. Many studies confirmed the singularities of the higher education students in what concerns food literacy and eating behaviors in different countries: Bangladesh [67], Belgium [68], China [69], Croatia [70], Egypt [70], Greece [70], Hungary [71], Italy [70], Jordan [72], Korea [73], Poland [74], Portugal [70,75], Serbia, [76], Slovenia [70], Spain [56,77], Syria [78], and the United States [79].

Additionally, this work resulted in a validation of some items that can in fact comprise the food literacy scale, against some others which were tested but that did not show enough consistency and a solid performance across the responses gathered in this survey. The internal consistency of a scale, either unifactorial or multifactorial, is determinant for its validity. Hence, from the 50 items tested, only about half (26 items) could actually be considered validated to make part of the scale. Reliability and validity are based on scale development and establishment. Reliability measures how dependently or consistently a specific measurement is achieved and encompasses different types of tests. For example, the internal consistency reliability indicates the extent to which items on a test measure the same thing, i.e., if the different items constitute a measurement cell [23,80,81].

With regards to the limitations, it is important to highlight the recruitment of participants, which followed a snowball methodology through internet tools on a convenience sample. As previously mentioned, the easy access, facility of recruitment, and readily verification of will to participate, along with the absence of funding requirement to conduct the survey, all contributed to the option of using a convenience sample, having in mind that studies have referred this as a useful approach to conduct exploratory research [13,14,15]. Convenience samples are frequently used in many studies reported on the scientific literature, for surveys about very different topics, precisely due to their advantages [82,83,84,85,86,87]. According to Brodaty et al. [88], who compared the performance of convenience versus population-based samples, the convenience sample of normal controls is likely to show better functioning. Still, possible sampling bias should be referred when interpreting the findings.

The use of convenience samples and internet snowball/cascade invitation results almost inevitably in uneven group representativeness [89]. For example, it is very frequent that more women than men participate in these surveys [82,90,91], because women are usually more willing to respond to questionnaire surveys. Nevertheless, there are also some studies in which the majority of participants are men [92,93].

Additionally, the data collection took place using online tools, while it is known that personal interviews could constitute a more feasible way for the data collection [15]. Nevertheless, the procedure would be incredibly more expensive, and it would also take more time to collect the data, and eventually it would not be possible to conduct the research without additional funding.

One other limitation is that this cross-sectional study cannot prove causality and can make the interpretation of the results challenging. Third, volunteer bias, such as recall bias and other types of bias, could have affected the study results [70].

As guidance for future work, it would be interesting to extend this research, now applying only the validated scale (with the final 26 items) to other population groups, like other student levels under university, or even groups of the general population and not only students. The food literacy scale constitutes an instrument that could be applied for example to compare responses from different countries as well, or from targeted groups: for example, according to the professional area (e.g., nutritionists *versus* other professionals) or behavioral variables, like physical activity or smoking habits.

## 5. Conclusions

The scientific literature is concomitant with the need to first investigate the levels of food literacy among all population groups, and with particular incidence in higher education students due to their particular vulnerability, on the one hand, and pivotal role in promoting healthy food habits. The use of validated scales is the most effective and correct way to assess the food literacy, and validation encompasses the need to follow established procedures that ensure statistical validity. The present scale has been validated for Portuguese higher education students, and can therefore be utilized from now on with guarantee of applicability. The validated scale comprises three factors, corresponding to three dimensions with most relevance, which have been discussed along this work, and which are as follows: F1—literacy about the nutritional composition of foods; F2—literacy about labelling and food choices; and F3—literacy about healthy eating practices. The validation model was a second-order model, with a global factor F4—food literacy. The number of items retained by the model was 26 (10 in subscale F1, 7 in subscale F2, and 9 in subscale F3). The internal consistency was very good in all subscales (the lowest value of Cronbach’s alpha being 0.897 and the highest being 0.931). Regarding the scale, reliability analysis also revealed a very high internal consistency, with a value of alpha equal to 0.962. Structural equation modelling was successful with good fitting indices: low value of square/degrees of freedom (3.702); values of CFI and GFI close to 1 (0.904 and 0.847, respectively); values of RMSEA, RMR, and SRMR are practically 0 (0.076, 0.054, and 0.055, respectively). The results from the Pearson correlations performed among the different factors and with the global model indicate very strong and significant correlations, revealing associations between these variables, with values of R varying from 0.725 to 0.954.

## Figures and Tables

**Table 1:** Reference values of the quality indicators for the adjustment of the model [28,29].

Statistic Indices	Reference Values
Qui-square/degrees of freedom	Value > 5—unacceptable fit Value (2; 5)—acceptable fit Value (1; 2)—good fit Value < 1—very good fit
CFI (Comparative Fit Index) and GFI (Goodness-of-Fit Index)	Value < 0.8—unacceptable fit Value (0.8; 0.9)—acceptable fit Value (0.9; 0.95)—good fit Value ≥ 0.95—very good fit
RMSEA (Root Mean Square Error of Approximation)	Value > 0.10—unacceptable fit Value (0.05; 0.10)—good fit Value ≤ 0.05—very good fit
RMR (Root Mean Square Residual)	Models with good fit have small values of RMR The lower the value, the better if the fit A perfect fit has RMR = 0
SRMR (Standardized Root Mean Square Residual)	Values ≤ 0.08 are desirable Value < 0.10 indicates an adequate fit SRMR = 0 indicates a perfect fit

**Table 2:** Age of the participants according to sex.

Sex	*n*	Minimum (Years)	Maximum (Years)	Mean	Standard Deviation	Coefficient of Variation
Female	736	18	57	21.89	5.37	24.53%
Male	188	18	70	24.14	8.17	33.84%

**Table 3:** Age group and type of course frequented according to sex.

Variables	Women		Men		Total	
	*n*	%	*n*	%	*n*	%
**Age**						
≤19 years	266	28.8	47	5.1	313	33.9
20–21 years	240	26.0	55	6.0	295	31.9
≥22 years	230	24.9	86	9.3	316	34.2
**Type of course**						
CTESP ^1^	5	0.5	14	1.5	19	2.1
License degree (graduation)	574	62.1	119	12.9	693	75.0
Master degree	123	13.3	38	4.1	161	17.4
Other	34	3.7	17	1.8	51	5.5
**Global**	**736**	**79.7**	**188**	**20.3**	**924**	**100.0**

^1^ CTESP = Higher Specialization Course [Curso Técnico Superior Especializado].

**Table 4:** Statistics relatively to all scale items and and internal consistency.

*n*	Item	Mean ^1^	Standard Deviation	Correlation Item-Total	Cronbach’s Alpha ^2^
1	Find information on healthy eating (website of the General Health Directory—DGS)—official Portuguese website)	3.92	1.28	0.472	0.981
2	Find information on healthy eating (internet; television; books/magazines)	4.36	0.88	0.523	0.981
3	Understand information on healthy eating (DGS website)	3.95	1.24	0.502	0.981
4	Understand information on healthy eating (internet; television; books/magazines)	4.28	08.4	0.657	0.980
5	Find information on daily meal frequency	4.15	09.6	0.621	0.980
6	Understand the importance of eating several times a day	4.37	0.83	0.542	0.981
7	Eat the recommended meals throughout the day, such as always having breakfast	4.18	09.1	0.433	0.981
8	Find information on diets and regimes (calorie restriction; vegetarian/vegan; organic; diets suitable for certain diseases/intolerances (e.g., gluten-free))	3.99	1.02	0.605	0.980
9	Understand the information about diets found on the internet	4.05	09.7	0.686	0.980
10	Understand the information about diets found in books/magazines	3.95	1.09	0.652	0.980
11	Practice a diet that fits the principles of healthy eating	3.77	0.99	0.642	0.980
12	Change your diet to a diet or diet suitable for certain diseases/intolerances	3.59	1.10	0.614	0.980
13	Find information on recommended portion sizes for each type of food	3.95	1.03	0.696	0.980
14	Understand recommendations on the amounts of food that should be consumed, when presented in portions	3.98	0.99	0.758	0.980
15	Understand the recommendations on the amounts of food that should be consumed, when presented in mass (grams)	3.90	1.06	0.762	0.980
16	Use the information to confirm that in your daily practice you consume the recommended amounts for your age and sex of the main food groups	3.70	1.09	0.719	0.980
17	Understand the information contained in the Portuguese Food Wheel	4.35	0.88	0.714	0.980
18	Find information on the nutritional quality of beverages	4.02	1.02	0.715	0.980
19	Understand the warnings about sugary drinks/soda consumption	4.32	0.93	0.686	0.980
20	Understand information about energy drinks (such as Red Bull)	4.17	1.06	0.670	0.980
21	Use the information to match your daily fluid intake needs	4.10	0.98	0.738	0.980
22	Find information about the Mediterranean Diet	3.91	1.19	0.728	0.980
23	Understand the assumptions of the Mediterranean Diet	3.83	1.21	0.732	0.980
24	Practice eating habits that conform to the standards of the Mediterranean Diet	3.65	1.20	0.677	0.980
25	Understand the benefits and risks associated with taking dietary supplements	3.88	1.16	0.718	0.980
26	Understand the usefulness of taking food supplements (Multivitamins, vitamins, calcium, Omega 3, etc.)	3.94	1.12	0.713	0.980
27	Find information on the use of salt in food	4.21	0.98	0.780	0.980
28	Understand the risks of excessive use of salt in food	4.30	0.94	0.776	0.980
29	Use the information to control the use of salt in your diet	4.10	1.03	0.751	0.980
30	Find information on the role of fats in food	4.20	0.99	0.782	0.980
31	Find information on the differences between saturated and unsaturated fats	3.94	1.12	0.807	0.980
32	Understand the effects resulting from the consumption of saturated and unsaturated fats	3.97	1.14	0.781	0.980
33	Moderate the use of fat in your diet	3.92	1.02	0.753	0.980
34	Avoid the use of saturated fats in your diet	3.83	1.07	0.734	0.980
35	Find information on the role of carbohydrates in food (rice; potato, pasta…)	4.21	1.00	0.778	0.980
36	Understand the type of carbohydrates you eat in your diet	4.01	1.05	0.797	0.980
37	Find information on the role of dietary fiber in health	4.09	1.05	0.806	0.980
38	Understand the benefits of consuming dietary fiber	4.09	1.05	0.786	0.980
39	Understand the benefits or drawbacks of excessive consumption of dietary fiber	3.96	1.11	0.792	0.980
40	Practice a diet that contains the recommended amounts of dietary fiber	3.77	1.08	0.778	0.980
41	Find information on the importance of adequate protein consumption in food (meat; fish; beans…)	4.13	1.03	0.797	0.980
42	Find information on animal and plant protein sources	4.08	1.05	0.808	0.980
43	Understand the recommended amounts for protein intake	3.93	1.15	0.812	0.980
44	Understand the effects of excessive protein consumption	3.90	1.16	0.765	0.980
45	Moderate your protein intake	3.81	1.06	0.730	0.980
46	Find information on how to interpret food labels	3.93	1.12	0.795	0.980
47	Understand the information conveyed on food labels	3.90	1.06	0.757	0.980
48	Find information about the nutritional semaphore on food labels	3.55	1.37	0.666	0.980
49	Understand the nutritional semaphore on food labels	3.57	1.36	0.633	0.981
50	Use food labelling to help make healthier food choices	3.84	1.10	0.694	0.980

^1^ Scale: 1 = Very difficult, 2 = Difficult, 3 = Easy, 4 = Very easy, 5 = Do not know. ^2^ Cronbach’s alpha without the item.

**Table 5:** Distributions of the items by the factors.

Factor Number and Name	Item Number (Corresponding Input Weight)
**F1** Literacy about the nutritional composition of foods	**17** (0.602), **18** (0.460), **19** (0.633), **20** (0.573), **21** (0.571), **25** (0.520), **26** (0.586), **27** (0.745), **28** (0.755), **29** (0.700), **30** (0.781), **31** (0.650), **32** (0.656), **33** (0.625), **34** (0.527), **35** (0.770), **36** (0.724), **37** (0.740), **38** (0.744), **39** (0.708), **40** (0.561), **41** (0.733), **42** (0.721), **43** (0.654), **44** (0.602), **45** (0.547)
**F2** Literacy about food labelling	**12** (0.478), **15** (0.553), **16** (0.596), **22** (0.522), **23** (0.558), **24** (0.573), **46** (0.576), **47** (0.547), **48** (0.775), **49** (0.772), **50** (0.605)
**F3** Literacy about healthy eating practices	**1** (0.411), **2** (0.612), **3** (0.479), **4** (0.709), **5** (0.683), **6** (0.655), **7** (0.594), **8** (0.657), **9** (0.718), **10** (0.619), **11** (0.519), **13** (0.533), **14** (0.545)

**Table 6:** Critical ratios and lambda coefficients of the items in the scale.

Items	Estimate (Lambda Coefficient)	Standard Error	Critical Ratio	*p*-Value
ba_17 ← F1	0.763	0.064	11.982	***
ba_18 ← F1	1.027	0.077	13.266	***
ba_19 ← F1	0.643	0.055	11.627	***
ba_20 ← F1	0.773	0.075	10.297	***
ba_21 ← F1	1.028	0.076	13.533	***
ba_25 ← F1	1.251	0.086	14.547	***
ba_26 ← F1	1.209	0.089	13.534	***
ba_27 ← F1	1.031	0.075	13.688	***
ba_28 ← F1	0.835	0.060	13.851	***
ba_29 ← F1	0.909	0.070	12.906	***
ba_30 ← F1	1.000			***
ba_31 ← F1	1.353	0.082	16.520	***
ba_32 ← F1	1.457	0.087	16.755	***
ba_33 ← F1	0.966	0.077	12.619	***
ba_34 ← F1	1.194	0.087	13.802	***
ba_35 ← F1	1.171	0.072	16.298	***
ba_36 ← F1	1.330	0.079	16.840	***
ba_37 ← F1	1.414	0.082	17.324	***
ba_38 ← F1	1.412	0.082	17.298	***
ba_39 ← F1	1.512	0.090	16.738	***
ba_40 ← F1	1.426	0.088	16.178	***
ba_41 ← F1	1.104	0.071	15.533	***
ba_42 ← F1	1.086	0.072	15.062	***
ba_43 ← F1	1.381	0.086	16.078	***
ba_44 ← F1	1.433	0.090	15.915	***
ba_45 ← F1	1.218	0.084	14.426	***
ba_12 ← F2	0.610	0.053	11.446	***
ba_15 ← F2	0.659	0.046	14.197	***
ba_16 ← F2	0.719	0.052	13.878	***
ba_22 ← F2	0.888	0.060	14.812	***
ba_23 ← F2	0.893	0.061	14.714	***
ba_24 ← F2	0.885	0.061	14.523	***
ba_46 ← F2	0.816	0.053	15.347	***
ba_47 ← F2	0.780	0.049	16.072	***
ba_48 ← F2	1.000			***
ba_49 ← F2	0.967	0.062	15.602	***
ba_50 ← F2	0.759	0.052	14.636	***
ba_01 ← F3	1.069	0.092	11.589	***
ba_02 ← F3	0.674	0.059	11.388	***
ba_03 ← F3	0.975	0.090	10.819	***

*** *p* < 0.001.

**Table 7:** Description of items of the second-order model.

*n*	Item	Parameters ^1^			
		Mean	SD	r	α
**F1**	**Literacy about the nutritional composition of foods**				
17	Understand the information contained in the Portuguese Food Wheel	104.99	194.47	0.679	0.961
18	Find information on the nutritional quality of beverages	105.26	190.98	0.722	0.961
21	Use the information to match your daily fluid intake needs	105.19	192.73	0.685	0.961
26	Understand the usefulness of taking food supplements (Multivitamins, vitamins, calcium, Omega 3, etc.)	105.23	191.39	0.722	0.961
31	Find information on the differences between saturated and unsaturated fats	105.22	190.25	0.767	0.961
32	Understand the effects resulting from the consumption of saturated and unsaturated fats	105.19	190.40	0.759	0.961
36	Understand the type of carbohydrates you eat in your diet	105.22	190.98	0.750	0.961
39	Understand the benefits or drawbacks of excessive consumption of dietary fiber	105.20	190.98	0.756	0.961
43	Understand the recommended amounts for protein intake	105.23	190.22	0.762	0.961
45	Moderate your protein intake	105.42	192.27	0.659	0.962
**F2**	**Literacy about labelling and food choices**				
15	Understand the recommendations on the amounts of food that should be consumed, when presented in mass (grams)	105.39	190.58	0.712	0.961
22	Find information about the Mediterranean Diet	105.17	191.05	0.727	0.961
24	Practice eating habits that conform to the standards of the Mediterranean Diet	105.35	192.32	0.633	0.962
46	Find information on how to interpret food labels	105.28	189.61	0.748	0.961
48	Find information about the nutritional semaphore on food labels	105.30	189.36	0.698	0.961
49	Understand the nutritional semaphore on food labels	105.31	189.95	0.683	0.961
50	Use food labelling to help make healthier food choices	105.39	191.00	0.667	0.962
**F3**	**Literacy about healthy eating practices**				
1	Find information on healthy eating (website of the General Health Directory—DGS)—official Portuguese website)	105.02	195.30	0.612	0.962
2	Find information on healthy eating (internet; television; books/magazines)	104.99	195.92	0.573	0.962
3	Understand information on healthy eating (DGS website)	105.04	195.00	0.635	0.962
5	Find information on daily meal frequency	105.13	193.44	0.677	0.961
6	Understand the importance of eating several times a day	105.01	195.73	0.588	0.962
8	Find information on diets and regimes (calorie restriction; vegetarian/vegan; organic; diets suitable for certain diseases/intolerances (e.g., gluten-free))	105.29	192.05	0.651	0.962
9	Understand the information about diets found on the internet	105.29	191.61	0.715	0.961
13	Find information on recommended portion sizes for each type of food	105.35	190.61	0.710	0.961
14	Understand recommendations on the amounts of food that should be consumed, when presented in portions	105.33	191.04	0.724	0.961

^1^ SD = Standard Deviation, r = Item–total correlation, α = Cronbach’s alpha without the item.

**Table 8:** Internal consistency of the items in the final model.

*n*	Item		Parameters ^1^			
		Mean	SD	r	r^2^	α
**F1**	**Literacy about the nutritional composition of foods**					**0.931**
17	Understand the information contained in the Portuguese Food Wheel	4.44	0.65	0.653	0.443	0.928
18	Find information on the nutritional quality of beverages	4.17	0.79	0.692	0.507	0.926
21	Use the information to match your daily fluid intake needs	4.24	0.73	0.681	0.483	0.927
26	Understand the usefulness of taking food supplements (multivitamins, vitamins, calcium, Omega 3, etc.)	4.20	0.76	0.700	0.495	0.926
31	Find information on the differences between saturated and unsaturated fats	4.21	0.78	0.795	0.733	0.921
32	Understand the effects resulting from the consumption of saturated and unsaturated fats	4.24	0.78	0.792	0.725	0.921
36	Understand the type of carbohydrates you eat in your diet	4.21	0.76	0.778	0.633	0.922
39	Understand the benefits or drawbacks of excessive consumption of dietary fiber	4.23	0.75	0.784	0.649	0.922
43	Understand the recommended amounts for protein intake	4.20	0.78	0.767	0.611	0.922
45	Moderate your protein intake	4.02	0.79	0.659	0.476	0.928
**F2**	**Literacy about labelling and food choices**					**0.897**
15	Understand the recommendations on the amounts of food that should be consumed, when presented in mass (grams)	4.04	0.81	0.603	0.392	0.892
22	Find information about the Mediterranean Diet	4.26	0.78	0.674	0.561	0.885
24	Practice eating habits that conform to the standards of the Mediterranean Diet	4.08	0.81	0.635	0.539	0.889
46	Find information on how to interpret food labels	4.15	0.82	0.721	0.567	0.879
48	Find information about the nutritional semaphore on food labels	4.13	0.89	0.770	0.764	0.873
49	Understand the nutritional semaphore on food labels	4.12	0.88	0.766	0.766	0.873
50	Use food labelling to help make healthier food choices	4.04	0.84	0.725	0.563	0.879
**F3**	**Literacy about healthy eating practices**					**0.908**
1	Find information on healthy eating (website of the General Health Directory—DGS—official Portuguese website)	4.42	0.67	0.680	0.504	0.898
2	Find information on healthy eating (internet; television; books/magazines)	4.44	0.68	0.650	0.504	0.900
3	Understand information on healthy eating (DGS website)	4.39	0.67	0.698	0.609	0.897
5	Find information on daily meal frequency	4.30	0.71	0.738	0.563	0.894
6	Understand the importance of eating several times a day	4.42	0.67	0.637	0.430	0.901
8	Find information on diets and regimes (calorie restriction; vegetarian/vegan; organic; diets suitable for certain diseases/intolerances (e.g., gluten-free))	4.14	0.81	0.682	0.537	0.898
9	Understand the information about diets found on the internet	4.15	0.76	0.730	0.592	0.894
13	Find information on recommended portion sizes for each type of food	4.08	0.82	0.696	0.629	0.897
14	Understand recommendations on the amounts of food that should be consumed, when presented in portions	4.10	0.78	0.672	0.617	0.898

^1^ SD = Standard Deviation, r = Item–total correlation, r^2^ = Correlation coefficient = Variance, α = Cronbach’s alpha without the item.

**Table 9:** Pierson’s correlation matrix between the scale factors.

	Factor F1	Factor F2	Factor F3	Factor global (F4)
**Factor F1**	1	0.849 ***	0.780 ***	0.954 ***
**Factor F2**		1	0.725 ***	0.921 ***
**Factor F3**			1	0.900 ***
**Factor Global (F4)**				1

*** Means a significant value at the 1% level (*p* < 0.01).

## Data Availability

Data are available from the corresponding author upon request.
